# The Use of Curcumin to Target Oxidative Stress and Inflammation in Type 2 Diabetes Mellitus and Its Complications: Molecular Mechanisms and Therapeutic Perspectives

**DOI:** 10.3390/antiox15081025

**Published:** 2026-08-17

**Authors:** Jia Zhang, Qipeng Shu, Yuntao Tang, Huilong Liu, Chenxi Zhang, Xiuhong Chen, Shangze Li

**Affiliations:** School of Medicine, Chongqing University, Chongqing 400030, China; 202437021014@stu.cqu.edu.cn (J.Z.); qipengshu@cqu.edu.cn (Q.S.); 202437021067t@stu.cqu.edu.cn (Y.T.); 202537105689@stu.cqu.edu.cn (H.L.); 202437021011@stu.cqu.edu.cn (C.Z.); chenxiuhong@stu.cqu.edu.cn (X.C.)

**Keywords:** type 2 diabetes mellitus, curcumin, oxidative stress, chronic inflammation, insulin resistance, molecular mechanism, adjuvant intervention

## Abstract

Type 2 diabetes mellitus (T2DM) is a chronic metabolic disorder characterized by insulin resistance, pancreatic β-cell dysfunction, and dysregulated glucose and lipid metabolism. Sustained hyperglycemia and hyperlipidemia promote excessive reactive oxygen species (ROS) production, antioxidant defense depletion, and chronic low-grade inflammation, thereby aggravating insulin signaling impairment, β-cell injury, and diabetes-related complications. Although current glucose-lowering therapies have improved glycemic control, weight management, and cardiorenal outcomes, oxidative stress and inflammation remain incompletely addressed in many individuals with T2DM. Curcumin, a natural polyphenol derived from Curcuma longa L., exhibits antioxidant, anti-inflammatory, lipid-regulating, insulin-sensitizing, and tissue-protective activities. Evidence suggests that curcumin may alleviate T2DM-associated oxidative stress by suppressing ROS generation, reducing nicotinamide adenine dinucleotide phosphate (NADPH) oxidase activity, modulating the advanced glycation end-product/receptor for advanced glycation end-product (AGE/RAGE) axis, activating nuclear factor erythroid 2-related factor 2/antioxidant response element (Nrf2/ARE) signaling, preserving mitochondrial homeostasis, and protecting β-cells. It may also inhibit nuclear factor-κB (NF-κB) and mitogen-activated protein kinase/c-Jun N-terminal kinase (MAPK/JNK) signaling, decrease pro-inflammatory cytokines and C-reactive protein (CRP), improve metabolic tissue inflammation, and attenuate gut-derived inflammation by regulating gut microbiota and intestinal barrier function. However, current clinical evidence mainly supports modest improvements in metabolic, inflammatory, oxidative stress-related, and selected complication-related biomarkers rather than definitive disease-modifying outcomes. Moreover, formulation heterogeneity, low bioavailability, limited pharmacokinetic reporting, and insufficient long-term endpoint data remain major translational barriers. This review summarizes the molecular mechanisms, clinical evidence, formulation-dependent interpretation, safety considerations, and translational limitations of curcumin as a candidate adjunctive intervention for T2DM, rather than as a replacement for evidence-based antidiabetic therapy.

## 1. Introduction

Type 2 diabetes mellitus (T2DM) is a chronic metabolic disease defined by insulin resistance, pancreatic β-cell dysfunction, and disturbances in glucose and lipid homeostasis. According to data from the International Diabetes Federation (IDF), approximately 589 million adults aged 20–79 years worldwide were living with diabetes in 2024, and this figure is projected to rise to 853 million by 2050 [1]. T2DM accounts for 90–95% of all diabetes cases and represents the primary driver of the growing global diabetes burden [2]. Long-term chronic hyperglycemia causes multi-tissue and multi-organ damage, leading to chronic complications such as diabetic nephropathy, retinopathy, peripheral neuropathy, and atherosclerotic cardiovascular disease—major contributors to diabetes-related disability and mortality [3]. Therefore, elucidating the key pathological mechanisms driving T2DM onset and progression, and exploring safe and effective adjuvant intervention strategies, are of great significance for delaying disease progression and reducing complication risk.

The development of T2DM is not attributable to elevated blood glucose alone; it arises from the interplay of multiple pathological processes including insulin resistance, β-cell functional decline, lipotoxicity, oxidative stress, and chronic low-grade inflammation [4]. Persistent exposure to high glucose and high lipid levels promotes excessive reactive oxygen species (ROS) production through mitochondrial electron transport chain dysfunction, polyol pathway activation, protein kinase C activation, upregulated NADPH oxidase expression, and activation of the advanced glycation end-product/receptor for advanced glycation end-product (AGEs/RAGE) axis [5,6,7]. Excess ROS directly damages pancreatic β-cells and peripheral tissues, including the liver, adipose tissue, skeletal muscle, and vascular endothelium, resulting in mitochondrial dysfunction, enhanced lipid peroxidation, and oxidative damage to proteins and DNA, which further aggravate insufficient insulin secretion and peripheral insulin resistance [8].

Oxidative stress and chronic inflammation operate in a mutually reinforcing cycle. ROS accumulation activates inflammation-related signaling pathways such as c-Jun N-terminal kinase (JNK), nuclear factor-κB (NF-κB), and the NOD-like receptor family pyrin domain-containing 3 (NLRP3) inflammasome, inducing the release of inflammatory mediators including tumor necrosis factor-α (TNF-α), interleukin-6 (IL-6), interleukin-1β (IL-1β), and monocyte chemoattractant protein-1 (MCP-1) [9]. In turn, pro-inflammatory cytokines upregulate NADPH oxidase expression, exacerbate mitochondrial damage, and inhibit insulin receptor substrate (IRS)/phosphoinositide 3-kinase (PI3K)/protein kinase B (Akt) signaling and glucose transporter type 4 (GLUT4) translocation [10]. This forms a positive feedback loop among ROS accumulation, enhanced inflammatory response, impaired insulin signaling, and β-cell functional decline.

**Figure 1:**
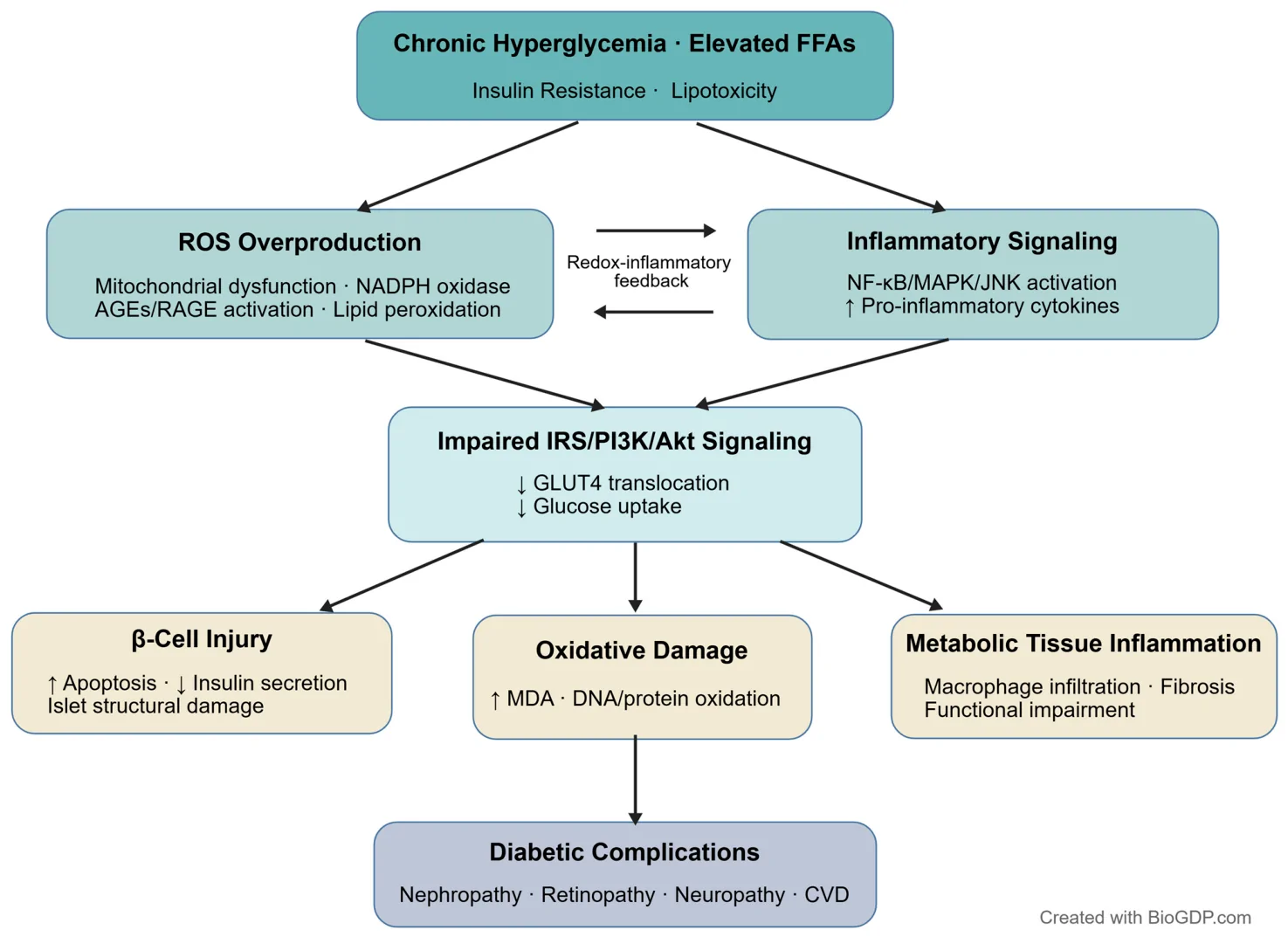
Core pathological network of oxidative stress and chronic inflammation in type 2 diabetes mellitus. Chronic hyperglycemia and elevated free fatty acids (FFAs) promote ROS overproduction and inflammatory signaling activation. ROS derived from mitochondrial dysfunction, NADPH oxidase activation, AGE/RAGE signaling, and lipid peroxidation interacts with NF-κB/MAPK/JNK-mediated inflammatory signaling and pro-inflammatory cytokine release, forming a redox–inflammatory feedback loop. These processes impair IRS/PI3K/Akt signaling, reduce GLUT4 translocation and glucose uptake, and contribute to β-cell injury, oxidative damage, metabolic tissue inflammation, and diabetic complications, including nephropathy, retinopathy, neuropathy, and cardiovascular disease. Created with BioGDP.com [11]. Arrows indicate the direction of pathophysiological influence; the bidirectional arrow denotes reciprocal redox–inflammatory feedback. Colors distinguish initiating metabolic stress (green), core redox/inflammatory and insulin-signaling modules (blue), intermediate tissue-injury outcomes (yellow), and diabetic complications (purple); colors do not indicate evidence strength.

The oxidative–inflammatory network linking chronic metabolic stress to insulin signaling impairment and diabetic complications is summarized in Figure 1.

Currently, T2DM management is built on lifestyle modification and pharmacotherapy. Agents including metformin, insulin sensitizers, α-glucosidase inhibitors, dipeptidyl peptidase-4 (DPP-4) inhibitors, sodium–glucose cotransporter 2 (SGLT2) inhibitors, glucagon-like peptide-1 (GLP-1) receptor agonists, and insulin have provided important improvements in glycemic control, weight management, and selected cardiorenal outcomes [12,13,14]. Nevertheless, long-term T2DM progression is driven collectively by oxidative stress, chronic inflammation, lipotoxicity, mitochondrial dysfunction, and β-cell decline. Existing treatments still lack stable, well-validated adjuvant interventions targeting the oxidative–inflammatory network and its mutually amplifying pathological interplay with insulin resistance, β-cell damage, and complication development.

Although contemporary antidiabetic therapies, including metformin, insulin, GLP-1 receptor agonists, SGLT2 inhibitors, and dual GIP/GLP-1 receptor agonists, have substantially improved glycemic control, body weight management, and cardiorenal outcomes in selected patients, oxidative stress and chronic low-grade inflammation remain incompletely addressed in many individuals with T2DM. These processes are closely involved in insulin resistance, pancreatic β-cell dysfunction, endothelial injury, mitochondrial stress, and the progression of diabetic complications, but they are not always primary therapeutic targets or principal endpoints in conventional glucose-centered treatment strategies. Therefore, adjunctive approaches targeting the oxidative stress–inflammation network may provide complementary value when positioned appropriately within evidence-based diabetes management.

Natural bioactive compounds have attracted growing research interest for metabolic disease prevention and treatment in recent years, owing to their wide availability, diverse molecular targets, and favorable safety profiles [15,16]. Among natural polyphenols and phytochemicals with antioxidant and anti-inflammatory properties, resveratrol, quercetin, berberine, and curcumin have all been investigated for potential metabolic benefits in T2DM. Curcumin, the principal polyphenolic active constituent of Curcuma longa L. rhizomes, was selected as the focus of this review because it combines a long history of traditional medicinal use with a broad pharmacological profile, including antioxidant, anti-inflammatory, lipid-regulating, insulin-sensitizing, β-cell-protective, and gut microbiota-modulating activities [17]. In particular, curcumin has been widely investigated as a multi-target redox–inflammatory modulator in experimental diabetes models and clinical supplementation studies. Prior studies indicate that curcumin reduces ROS and lipid peroxidation product levels, enhances endogenous antioxidant enzyme activity, inhibits inflammation-related signaling such as NF-κB and MAPK/JNK, and improves selected markers of glucose metabolism, inflammation, and oxidative stress [18,19,20]. Nevertheless, poor aqueous solubility, low oral bioavailability, rapid metabolism, and formulation-dependent exposure remain major barriers to its clinical translation. Accordingly, novel delivery systems and formulation strategies, including nanoparticle-, liposome-, micelle-, phytosome-, and piperine-based approaches, have been developed to improve curcumin stability, absorption, and systemic exposure.

Although several reviews and meta-analyses have summarized the general antidiabetic effects of curcumin, the integrated relationship among oxidative stress, chronic inflammation, clinical evidence, and translational limitations in T2DM remains less fully addressed. Many previous reviews have mainly emphasized glycemic outcomes, broad pharmacological properties, or selected molecular pathways. In contrast, the present review is organized around the oxidative stress–inflammation network as a central pathological axis linking insulin resistance, pancreatic β-cell dysfunction, and diabetic complications. Furthermore, this review distinguishes preclinical mechanistic evidence from clinical biomarker-based findings and critically discusses formulation-related heterogeneity, bioavailability barriers, safety considerations, and the role of curcumin as an adjunctive intervention rather than a replacement for evidence-based antidiabetic therapy. This framework aims to clarify the current evidence base and identify key translational gaps for future standardized clinical studies. The main conceptual contribution of this review is to reframe curcumin not simply as a multi-target antioxidant or anti-inflammatory compound, but as a formulation-dependent adjunctive intervention candidate. From this perspective, the translational value of curcumin depends not only on whether it modulates ROS generation, Keap1/Nrf2/ARE signaling, NF-κB activation, or inflammatory cytokines, but also on whether standardized preparations can provide clinically measurable incremental redox–inflammatory benefits beyond evidence-based glucose-lowering and cardiorenal-protective therapy. This formulation- and endpoint-centered framework distinguishes the present review from previous broad summaries of curcumin’s antidiabetic mechanisms.

## 2. Literature Search Strategy

This narrative review was prepared with reference to the SANRA framework for the assessment of narrative review articles, with particular attention to justification of the review’s importance, clear statement of aims, transparent description of the literature search, appropriate referencing, scientific reasoning, and balanced presentation of endpoint data [21].

The literature search was last updated on 11 August 2026. PubMed, Web of Science, Scopus, and Google Scholar were searched using combinations of the following terms: (“curcumin” OR “turmeric” OR “curcuminoids” OR “Curcuma longa”) AND (“type 2 diabetes mellitus” OR “T2DM” OR “prediabetes” OR “insulin resistance”) AND (“oxidative stress” OR “chronic inflammation” OR “Nrf2” OR “Keap1/Nrf2/ARE” OR “NF-κB” OR “AGEs/RAGE” OR “MAPK” OR “JNK” OR “mitochondrial dysfunction” OR “gut microbiota” OR “diabetic complications” OR “bioavailability” OR “formulation”). Additional targeted searches were performed for specific complications and translational topics using terms including “diabetic nephropathy”, “diabetic retinopathy”, “diabetic neuropathy”, “cardiovascular complications”, “MASLD”, “diabetic foot ulcer”, “wound healing”, “curcumin-piperine”, “nanocurcumin”, “micellar curcumin”, “liposomal curcumin”, “phytosomal curcumin”, “pharmacokinetics”, “safety”, and “drug interaction”.

Approximately 1300 records were initially identified from PubMed, Web of Science, and Scopus before duplicate removal. After removal of duplicates and screening of titles and abstracts, approximately 256 articles were considered potentially relevant. Finally, 104 articles were included in the narrative synthesis based on mechanistic relevance, clinical applicability, recency, formulation specificity, and contribution to the translational scope of this review. Google Scholar was used mainly for supplementary targeted searches and cross-checking of highly relevant studies. The reference lists of key randomized controlled trials, systematic reviews, meta-analyses, pharmacokinetic studies, and formulation-related reviews were manually screened to identify additional relevant publications.

Article selection was performed independently by two authors. Disagreements regarding study relevance, evidence priority, or interpretation were resolved through discussion with the corresponding author. Priority was given to randomized controlled trials, systematic reviews, meta-analyses, pharmacokinetic studies, formulation-related studies, and recent original mechanistic studies directly related to curcumin, oxidative stress, inflammation, T2DM, diabetic complications, or formulation optimization. Foundational mechanistic studies were retained when they provided essential biological context. Recent clinical and translational evidence was prioritized when available, particularly for clinical outcomes, formulation-specific interpretation, bioavailability, safety, and drug interactions.

Because this article is a narrative review rather than a systematic review or meta-analysis, a formal PRISMA-based selection process, quantitative synthesis, and formal risk-of-bias scoring were not performed. However, the strength of evidence was considered according to study type, sample size, intervention duration, formulation characteristics, consistency across studies, clinical relevance of endpoints, and whether outcomes were based on preclinical mechanisms, surrogate biomarkers, or clinically meaningful events.

## 3. Current Therapeutic Landscape of Type 2 Diabetes Mellitus and Unmet Needs for Oxidative–Inflammatory Intervention

### 3.1. Conventional Therapeutic Strategies

T2DM management is typically founded on lifestyle intervention, with pharmacotherapeutic regimens individualized based on glycemic control, weight status, hypoglycemia risk, cardiovascular disease, chronic kidney disease, and patient-specific characteristics [13,22]. Early intensive glycemic control remains a cornerstone of T2DM care. Long-term follow-up of the UK Prospective Diabetes Study (UKPDS) demonstrated that intensive therapy with sulfonylureas or insulin in newly diagnosed T2DM patients reduced risks of all-cause mortality, myocardial infarction, and microvascular disease. In overweight patients, metformin treatment likewise reduced all-cause mortality and myocardial infarction risk, and the associated “metabolic memory” or “legacy effect” remained stable over extended follow-up [23,24].

In pharmacotherapy, metformin is generally recommended as first-line and foundational treatment. Subsequent combination or switching to sulfonylureas, insulin, DPP-4 inhibitors, GLP-1 receptor agonists, SGLT2 inhibitors, and other agents is guided by glycemic response and comorbid conditions [13]. In recent years, SGLT2 inhibitors and long-acting GLP-1 receptor agonists have demonstrated benefits across cardiovascular events, heart failure hospitalization, kidney disease progression, and all-cause mortality, gradually shaping a comprehensive T2DM care paradigm centered on glycemic control with integrated weight management and cardiorenal protection [25,26,27].

It should be acknowledged that several modern antidiabetic agents may exert indirect anti-inflammatory and antioxidant effects. In particular, GLP-1 receptor agonists and SGLT2 inhibitors have been reported to improve inflammatory status, oxidative stress-related biomarkers, endothelial function, mitochondrial stress, and cardiorenal metabolic homeostasis in selected experimental and clinical contexts. However, these effects are generally considered pleiotropic or secondary consequences of improved metabolic control, weight reduction, hemodynamic regulation, and cardiorenal protection, rather than their primary therapeutic targets. Moreover, oxidative stress and chronic low-grade inflammation are not consistently assessed as principal endpoints in conventional glucose-centered treatment trials. Therefore, despite major advances in contemporary pharmacotherapy, the oxidative stress–inflammation network remains an incompletely addressed pathological axis in T2DM.

### 3.2. Limitations of Current Therapies in Targeting Oxidative Stress and Chronic Inflammation

Although existing therapeutic strategies have markedly improved glycemic control, body weight management, and selected cardiorenal outcomes in T2DM, the disease involves multi-layered pathological processes, including insulin resistance, β-cell dysfunction, lipid metabolism abnormalities, endothelial injury, mitochondrial stress, vascular damage, oxidative stress, and chronic low-grade inflammation [13]. As discussed above, some modern antidiabetic agents may indirectly modulate oxidative–inflammatory pathways; however, most clinical trials of glucose-lowering drugs prioritize metabolic or hard clinical endpoints such as HbA1c, body weight, major adverse cardiovascular events, heart failure hospitalization, kidney disease progression, and mortality. Oxidative stress and chronic inflammation are generally not primary therapeutic targets or core evaluation metrics.

Oxidative stress and chronic inflammation are fundamental pathological underpinnings of T2DM and its metabolic complications and are associated with abnormalities in oxidative and inflammatory markers such as malondialdehyde (MDA), TNF-α, high-sensitivity C-reactive protein (hs-CRP), and MCP-1 [28]. Phytochemicals have been shown to improve selected oxidative–inflammatory markers and modulate glucose and lipid metabolism, but findings remain inconsistent across studies [29]. Accordingly, exploring complementary or adjunctive intervention strategies targeting oxidative stress and chronic low-grade inflammation as an add-on to standard therapy remains a valuable research direction.

### 3.3. Rationale for Curcumin as a Candidate Adjuvant Intervention

With its multifaceted pharmacological profile, including antioxidant, anti-inflammatory, lipid-regulating, insulin-sensitizing, β-cell-protective, and gut microbiota-modulating activities, curcumin has been proposed as a potential complementary intervention candidate in T2DM when added to standard care [22,30]. Systematic reviews and animal experiments indicate that curcumin may improve fasting blood glucose, HbA1c, BMI, and selected lipid and inflammatory–oxidative markers, and may delay diabetes onset, preserve pancreatic β-cell function, and reduce insulin resistance.

Overall, available clinical evidence suggests that curcumin may favorably affect selected markers of glycemic control, inflammatory status, and oxidative stress in T2DM. A meta-analysis of 18 trials enrolling 1382 T2DM patients found that curcumin supplementation reduced fasting blood glucose, HbA1c, and C-reactive protein [31]. Other studies report that curcumin or turmeric supplementation lowers CRP, TNF-α, IL-6, and MDA while increasing GSH and total antioxidant capacity, although the certainty and generalizability of these findings are limited by study heterogeneity [32].

The comparison between curcumin and contemporary T2DM pharmacotherapy should not be framed as a competition in glucose-lowering efficacy. Evidence-based therapies, including metformin, GLP-1 receptor agonists, SGLT2 inhibitors, and tirzepatide, provide clinically meaningful metabolic, cardiovascular, or renal benefits in appropriate patient populations and are incorporated into current diabetes treatment algorithms [25,26,27,33,34]. Importantly, some of these agents may also influence inflammatory and redox biology directly or indirectly through improvements in glycemia, body weight, adiposity, endothelial function, hemodynamics, mitochondrial stress, and cardiorenal metabolism [35,36]. In contrast, clinical studies of curcumin mainly report changes in surrogate metabolic, inflammatory, and oxidative stress-related biomarkers, with limited evidence for long-term clinical outcomes, including diabetic complications, cardiovascular events, renal outcomes, mortality, and quality of life [31,32,37,38,39]. Therefore, the clinically relevant question is not whether curcumin can replace evidence-based pharmacotherapy, but whether standardized curcumin formulations can provide measurable incremental redox–inflammatory benefits when added to established treatment, such as metformin, GLP-1 receptor agonists, or SGLT2 inhibitors. Future adjunctive trials should evaluate whether curcumin provides additive benefits beyond background therapy, using predefined oxidative stress and inflammatory endpoints, pharmacokinetic assessment, and clinically meaningful metabolic or complication-related outcomes.

## 4. Pharmacological Profile of Curcumin and the Basis of Its Antidiabetic Actions

### 4.1. Physicochemical and Pharmacokinetic Properties

Curcumin, also known as diferuloylmethane, is the dominant curcuminoid in turmeric rhizomes. A natural polyphenolic compound, it exhibits antioxidant, anti-inflammatory, lipid-regulating, immunomodulatory, and antidiabetic activities [30]. Its phenolic hydroxyl groups, β-diketone moiety, and conjugated double-bond system provide the structural basis for free radical scavenging, redox modulation, and multi-target signal regulation. However, clinical translation of curcumin is hampered by poor aqueous solubility, chemical instability, limited intestinal absorption, rapid in vivo metabolism, and low systemic exposure [40,41].

Following oral administration, curcumin’s high hydrophobicity and low aqueous solubility limit its dissolution in the gastrointestinal tract and subsequent transepithelial absorption. The fraction that is absorbed undergoes extensive phase II metabolism including glucuronidation and sulfation, resulting in low plasma concentrations of free curcumin [40,42]. In addition, curcumin metabolites, including glucuronidated, sulfated, and reduced derivatives, may retain partial biological activity or contribute to local gastrointestinal effects. Therefore, both parent curcumin and its metabolites should be considered when interpreting pharmacological outcomes. Accordingly, improving solubility, stability, mucosal absorption, and target tissue delivery is critical to enhancing the reproducibility of its antidiabetic effects. Delivery systems including nanoparticles, liposomes, chitosan carriers, protein carriers, and protein–polysaccharide complexes have been developed to address the pharmacokinetic shortcomings of curcumin [41,43,44]. Compared with conventional unformulated curcumin, newer formulations such as phytosomal, nanoparticle-based, micellar, and liposomal preparations generally aim to improve aqueous dispersion, intestinal absorption, systemic exposure, and tissue delivery. However, improved pharmacokinetic exposure does not necessarily indicate superior clinical efficacy, and head-to-head clinical comparisons among different formulations remain limited. Pharmacokinetic variability is another important factor limiting the reproducibility of curcumin-related clinical effects. Oral curcumin exposure may be influenced by differences in gastrointestinal dissolution, intestinal permeability, gut microbiota composition, hepatic and intestinal phase II metabolism, dietary background, and concomitant medications. Because curcumin undergoes rapid glucuronidation and sulfation, circulating concentrations of free curcumin are often low and may vary substantially among individuals. Therefore, the biological effects observed after supplementation may depend not only on the administered dose, but also on formulation type, absorption efficiency, metabolic conversion, local intestinal exposure, and tissue distribution.

### 4.2. Antioxidant, Anti-Inflammatory, and Metabolic Regulatory Foundations

Oxidative stress and chronic low-grade inflammation are central pathological drivers of T2DM onset, progression, and complication development. The antioxidant and anti-inflammatory activities of curcumin form the primary pharmacological basis for its antidiabetic potential [28,45]. In terms of antioxidant activity, curcumin scavenges reactive oxygen species, enhances antioxidant enzyme activity, reduces lipid peroxidation damage, and helps maintain redox homeostasis. With respect to anti-inflammatory activity, curcumin modulates inflammation-related transcription factors and signaling pathways, including NF-κB, STAT, AP-1, and MAPK, and regulates the expression of inflammatory mediators, including TNF-α, IL-1β, IL-6, and MCP-1 [40,46].

The antidiabetic effect of curcumin does not originate from a single hypoglycemic target, but rests on its multi-level modulation of oxidative stress, chronic inflammation, insulin resistance, β-cell damage, lipid metabolism dysregulation, and complication-related pathological processes [22,47]. Preclinical and clinical studies indicate that curcumin improves β-cell function, insulin resistance, fasting blood glucose, HbA1c, and C-reactive protein, and ameliorates inflammatory and oxidative stress markers in T2DM patients with metabolic dysfunction-associated steatotic liver disease (MASLD) [31,48]. Accordingly, curcumin is best conceptualized as a multi-target metabolic regulator, with antioxidant and anti-inflammatory actions as central features and broader effects on glucose-lipid metabolism and target-organ protection.

## 5. Molecular Mechanisms Underlying Curcumin Regulation of Oxidative Stress in Type 2 Diabetes Mellitus

Oxidative stress is a core pathological process in the development of T2DM and its complications. Long-term hyperglycemia, elevated free fatty acids, and insulin resistance lead to increased ROS production, depletion of antioxidant defenses, mitochondrial dysfunction, enhanced lipid peroxidation, and protein glycation damage, which in turn accelerate pancreatic β-cell functional decline, impaired insulin signaling, and tissue inflammation. Available evidence indicates that the antidiabetic effects of curcumin are not solely dependent on glucose lowering, but are closely linked to inhibiting ROS generation, reducing lipid peroxidation products such as MDA, enhancing antioxidant defenses (SOD, GSH, GPx, CAT), modulating the AGE/RAGE axis, activating Nrf2/ARE signaling, and preserving mitochondrial and β-cell function [29,30,32]. It should be noted that much of the pathway-level evidence discussed in this section is derived from in vitro experiments and animal models of T2DM or diabetes-related organ injury. Clinical studies have mainly reported changes in systemic oxidative stress biomarkers, such as MDA, TAC, SOD, GPx, and GSH, rather than direct confirmation of pathway activation or inhibition in human target tissues. Therefore, the following mechanisms should be interpreted as biologically plausible and experimentally supported pathways that require further validation in human T2DM populations. Although oxidative stress and inflammation are closely interconnected in T2DM, this section focuses primarily on ROS generation, antioxidant defense, mitochondrial dysfunction, pancreatic β-cell oxidative injury, and insulin signaling impairment. Inflammatory pathways such as NF-κB and MAPK/JNK are discussed here only when they function as downstream mediators of oxidative stress-related metabolic injury.

### 5.1. Inhibition of ROS Production and Modulation of the AGE/RAGE Axis

In T2DM, chronic hyperglycemia and glucolipotoxicity drive excess ROS production via increased mitochondrial electron transport chain load, NADPH oxidase activation, AGE accumulation, and RAGE engagement. ROS accumulation not only inflicts direct oxidative damage to lipids, proteins, and nucleic acids, but also activates signaling networks including NF-κB, JNK, PKC, and PI3K/Akt, triggering inflammatory cytokine release, insulin signaling impairment, and diabetic complication progression [49]. Therefore, suppressing ROS sources and blocking AGE/RAGE-mediated oxidative–inflammatory amplification represent key mechanisms by which curcumin regulates oxidative stress in T2DM.

The AGE/RAGE axis is a critical pathway linking oxidative stress, inflammation, and tissue fibrosis in the hyperglycemic milieu. In diabetes, AGE–RAGE binding induces ROS generation and inflammatory signaling activation, and promotes injury to target organs including the liver, kidney, vasculature, and pancreatic islets. In vitro studies show that AGEs upregulate RAGE expression and activate hepatic stellate cells, whereas curcumin attenuates AGE-induced oxidative stress by enhancing PPARγ activity and promoting de novo glutathione synthesis, and further downregulates RAGE gene expression, thereby blunting AGE-stimulated hepatic stellate cell activation [50]. These findings suggest that beyond direct free radical scavenging, curcumin interrupts hyperglycemia-driven oxidative stress amplification by regulating upstream AGE/RAGE receptor expression and boosting intracellular antioxidant capacity.

In a mouse model of HFD/STZ-induced T2DM with liver injury, network pharmacology and molecular docking analysis implicated the AGE-RAGE signaling pathway in the hepatoprotective effects of curcumin, with strong binding affinity predicted between curcumin and RAGE, AKT1, and TP53. In vivo experiments further confirmed that curcumin improved hyperglycemia, reduced hepatic oxidative stress and inflammation, inhibited the AGE-RAGE pathway, and modulated downstream PI3K/Akt and NF-κB signaling [51]. This study connects the AGE-RAGE axis to PI3K/Akt, NF-κB, and metabolomic alterations. These preclinical findings suggest that curcumin may attenuate T2DM-related liver injury partly through modulation of the AGE-RAGE axis and its downstream oxidative–inflammatory signaling. However, direct evidence that curcumin suppresses AGE/RAGE signaling in human T2DM target tissues remains limited.

Inhibition of ROS production is also tightly associated with reduced NADPH oxidase activity. Pancreatic β-cells have relatively weak antioxidant defenses and are highly susceptible to ROS damage induced by glucolipotoxicity. Studies in INS-1 cells and STZ-induced diabetic rats demonstrated that high glucose/palmitic acid triggered ROS generation, upregulated NADPH oxidase subunits, decreased antioxidant enzyme activity, and increased apoptotic factors. Curcumin treatment suppressed oxidative stress activation, downregulated NADPH oxidase subunits and apoptosis-related molecules, increased insulin levels, and mitigated histopathological damage to pancreatic islets [52]. Clinical studies likewise show that T2DM patients supplemented with curcuminoids plus piperine exhibit elevated serum total antioxidant capacity (TAC) and SOD activity alongside reduced MDA levels [53]. Taken together, curcumin regulates ROS both by scavenging existing ROS and potentially by reducing NADPH oxidase-derived ROS generation.

### 5.2. Activation of Keap1/Nrf2/ARE-Mediated Antioxidant Defense

Nuclear factor erythroid 2-related factor 2 (Nrf2) is the master transcription factor governing cellular antioxidant responses. Under basal conditions, Nrf2 is targeted for ubiquitination and degradation by Kelch-like ECH-associated protein 1 (Keap1). Under oxidative stress, Nrf2 dissociates from Keap1, translocates to the nucleus, and binds to antioxidant response elements (AREs), driving the expression of antioxidant and phase II detoxifying enzymes, including heme oxygenase-1 (HO-1), NAD(P)H quinone oxidoreductase 1 (NQO1), glutamate-cysteine ligase catalytic subunit (GCLC), catalase (CAT), superoxide dismutase (SOD), and glutathione peroxidase (GPx). In diabetes, persistent ROS elevation depletes antioxidant defenses, and activation of the Nrf2/ARE axis is a key mechanism for restoring redox homeostasis [54]. Hence, enhancement of endogenous antioxidant defense via the Nrf2/ARE pathway represents a major molecular basis for curcumin-mediated oxidative stress regulation in T2DM.

In a rat model of diabetic kidney disease, curcumin improved albuminuria and glomerular pathology, reduced urinary MDA levels, and mitigated renal lipid deposition and oxidative stress damage by amplifying Nrf2 signaling and activating AMPK signaling [55]. This result indicates that the renoprotective effect of curcumin in diabetes is not merely secondary to glucose lowering; it is at least partially mediated by Nrf2-driven restoration of antioxidant defense and alleviation of renal lipotoxicity. In a rat model of testicular oxidative stress in T2DM, curcumin and its analog J7 reduced MDA levels, increased SOD activity, and upregulated p-Nrf2/total Nrf2, CAT, NQO1, and HO-1 expression, supporting a role for Nrf2/ARE signaling restoration in tissue protection [56].

Regarding clinical evidence, direct measurements of Nrf2 pathway activation by curcumin remain limited, but improvements in Nrf2 downstream functional antioxidant markers show reasonable consistency. In T2DM patients, curcuminoid supplementation increases TAC and SOD activity and decreases MDA [53]; a meta-analysis of prediabetic and T2DM populations likewise reports that curcumin or turmeric supplementation reduces MDA while raising GSH and TAC [32]. In T2DM patients with metabolic dysfunction-associated steatotic liver disease (MASLD), 1500 mg/day curcumin for 12 months reduced MDA and increased GPx and SOD activity [48]. Notably, studies vary in dosage, formulation, intervention duration, disease model, and target organ. For this reason, Keap1/Nrf2/ARE should be presented as an important pathway in curcumin’s antioxidant mechanism, rather than as the sole or definitively proven target. Thus, although Nrf2/ARE activation is a plausible and well-supported mechanism in experimental models, its direct clinical relevance in human T2DM remains to be established through studies assessing pathway-specific markers in relevant tissues or validated circulating readouts. This interpretation also places curcumin within the broader class of redox-modulating phytochemicals. Keap1/Nrf2/ARE activation is not unique to curcumin, but represents a shared adaptive antioxidant mechanism triggered by several natural antioxidants and electrophilic compounds. Therefore, the relevance of curcumin lies less in pathway specificity than in its ability to modulate Nrf2/ARE signaling together with other oxidative–inflammatory targets.

### 5.3. Maintenance of Mitochondrial Homeostasis and Attenuation of Organelle Stress

Mitochondria are both a major source and a critical target of oxidative stress in T2DM. Long-term hyperglycemia, hyperlipidemia, and insulin resistance increase mitochondrial substrate oxidation load, causing electron transport chain leakage, elevated ROS production, disrupted mitochondrial membrane potential, impaired ATP synthesis, and cytochrome c release—events that ultimately drive apoptosis and tissue dysfunction. Beyond bioenergetic dysfunction and mitochondria-associated apoptosis, mitochondrial quality-control processes may also contribute to metabolic homeostasis in T2DM. These processes include mitochondrial biogenesis, fusion–fission dynamics, and mitophagy, which regulate mitochondrial turnover, stress adaptation, and metabolic flexibility. Although direct evidence that curcumin modulates these processes in T2DM remains limited, they represent relevant future directions for understanding curcumin-related mitochondrial protection. Studies in db/db mice show that hyperglycemia alters hepatic and renal mitochondrial oxygen consumption, NO synthesis, and lipid peroxidation status, whereas curcumin treatment normalizes or restores these mitochondrial functional parameters and increases hepatic mitochondrial ATPase activity, indicating a protective effect of curcumin against diabetes-related mitochondrial dysfunction [57].

In diabetic cardiomyopathy models, mitochondrial damage is closely linked to cardiomyocyte apoptosis, oxidative stress, and cardiac dysfunction. In diabetic rats induced by STZ plus a high-glucose, high-fat diet, elevated MDA, gp91phox, and cytochrome c were observed alongside increased Bax/Bcl-2 ratio, cleaved caspase-3, and TUNEL-positive cells. Curcumin ameliorated cardiac dysfunction, oxidative stress, and apoptosis, enhanced Akt phosphorylation, and inhibited FoxO1 acetylation [58]. A separate study of diabetic cardiomyopathy in db/db mice likewise found that curcumin suppressed ROS production, improved myocardial histopathology, and exerted cardioprotection by upregulating Bcl-2 and downregulating Bax and caspase-3 [59]. Collectively, these studies indicate that curcumin preserves mitochondrial homeostasis principally by reducing ROS generation, inhibiting lipid peroxidation, suppressing mitochondria-associated apoptotic signaling, and improving tissue structure and function.

Mitochondrial homeostasis in pancreatic β-cells is also tightly coupled to insulin secretion and cell survival. In a palmitic acid-induced lipotoxicity model of MIN6 β-cells, palmitic acid promoted ROS generation and suppressed antioxidant enzyme activity, while curcumin reversed these changes and induced rapid Akt phosphorylation and FoxO1 nuclear export. PI3K and Akt inhibitors abolished the anti-lipotoxic effects of curcumin and promoted FoxO1 nuclear translocation, indicating that β-cell protection by curcumin is dependent on Akt activation, FoxO1 regulation, and mitochondrial survival pathways [60].

With respect to endoplasmic reticulum stress, there remain relatively few T2DM curcumin studies using canonical markers such as PERK, IRE1α, ATF6, or CHOP as primary endpoints. At this stage, the most cautious conclusion is that curcumin may indirectly alleviate the organelle stress network linked to oxidative stress by improving mitochondrial function, reducing ROS levels, and inhibiting apoptotic signaling; however, its direct regulation of classic endoplasmic reticulum stress pathways requires further validation. Overall, the evidence linking curcumin to mitochondrial protection in T2DM is primarily preclinical. These studies support a potential role of curcumin in reducing mitochondrial ROS production, preserving mitochondrial membrane potential, and attenuating mitochondria-associated apoptosis. However, whether these effects translate into sustained mitochondrial functional improvement in human T2DM tissues has not been adequately established. Further studies should clarify whether curcumin directly regulates mitochondrial quality-control processes in T2DM.

### 5.4. Pancreatic β-Cell Protection and Improvement of Insulin Signaling

β-cell functional decline is a key driver of T2DM progression, and β-cells are highly vulnerable to oxidative stress. Glucolipotoxicity induces ROS generation, NADPH oxidase activation, mitochondrial damage, and enhanced apoptotic signaling, ultimately leading to insufficient insulin secretion and deteriorating glycemic control. Curcumin protects β-cells primarily by reducing oxidative damage, enhancing antioxidant enzyme activity, inhibiting apoptosis, preserving islet architecture, and improving insulin secretion [61,62].

In an STZ-induced T2DM rat model, curcumin reduced pancreatic MDA levels, restored GPx and SOD activity, decreased islet cell apoptosis, and increased insulin secretion, supporting β-cell survival and function preservation via antioxidant mechanisms [61]. Another T2DM rat study reported that curcumin lowered fasting blood glucose, promoted pancreatic functional recovery, reduced pancreatic tissue destruction and apoptotic index, and decreased IL-1β, IL-6, TNF-α, caspase-3, Bax, and MDA while increasing Bcl-2, SOD2, and GPx. Mechanistically, curcumin inhibited JNK and NF-κB phosphorylation, thereby blocking the RAGE/JNK/NF-κB signaling cascade and attenuating β-cell inflammation and apoptosis [62]. These findings support a β-cell-protective effect of curcumin in experimental T2DM models. However, whether such improvements in β-cell function are sustained over time remains uncertain, because most available evidence is derived from animal models or relatively short-term biomarker-based clinical observations rather than long-term assessments of β-cell preservation in humans.

Curcumin also participates in oxidative stress regulation in T2DM by improving insulin signal transduction. Hyperglycemia and ROS reduce IRS/PI3K/Akt pathway activity, impair GLUT4 translocation, and decrease tissue glucose uptake. In a T2DM liver injury model, curcumin inhibited the AGE-RAGE axis and modulated downstream PI3K/Akt signaling, suggesting it may improve insulin-related signaling networks while reducing oxidative–inflammatory damage [51]. In T2DM rats, high-dose curcumin reduced fasting blood glucose, serum lipids, hepatic MDA, and Bax expression, increased hepatic SOD, CAT, GSH, as well as PI3K, p-PI3K, Akt, and p-Akt levels, and improved hepatic and pancreatic histology [63].

**Figure 2:**
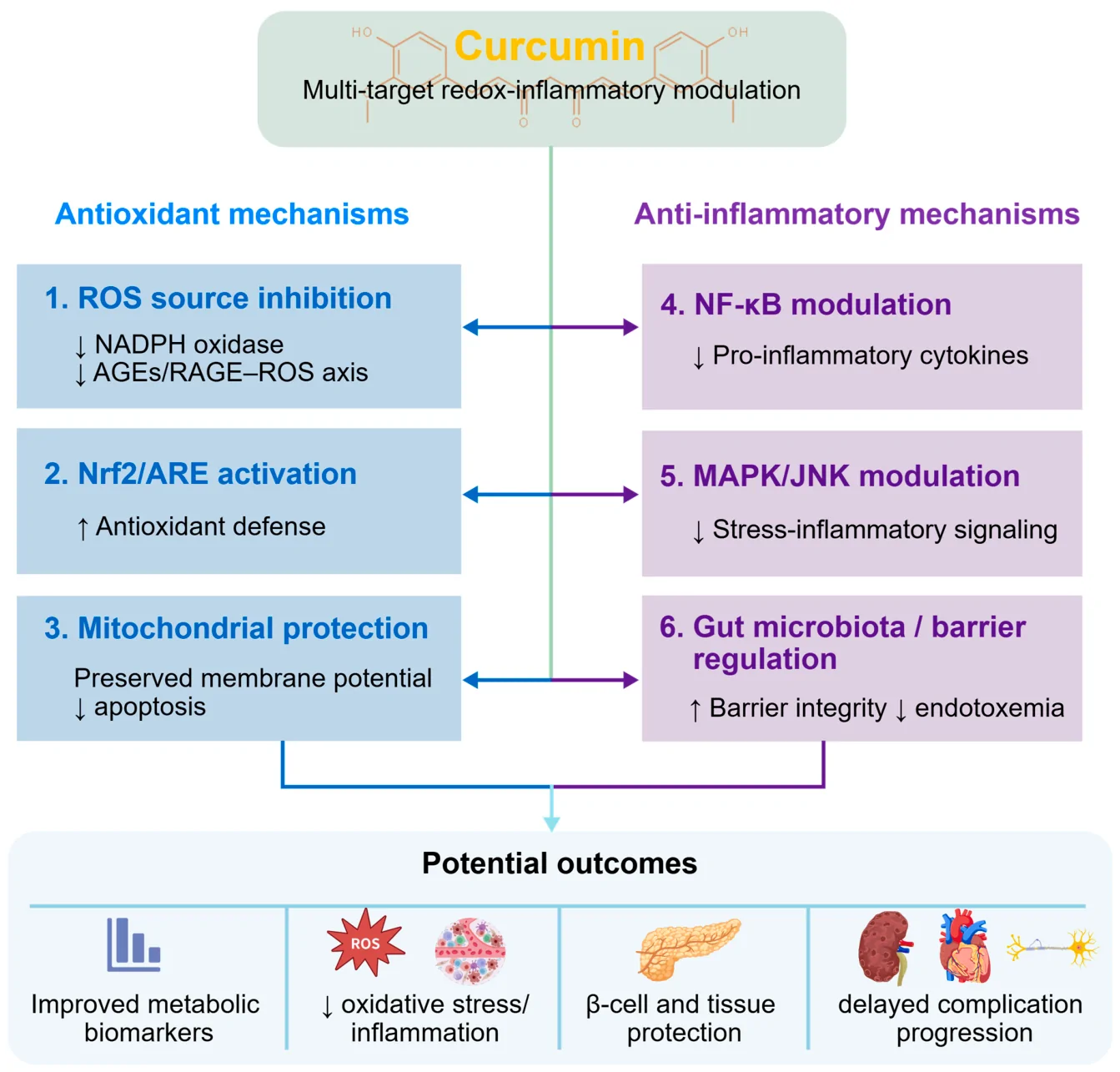
Integrated multi-target mechanism of curcumin in modulating oxidative stress and chronic inflammation in type 2 diabetes mellitus. Curcumin may act through both antioxidant and anti-inflammatory mechanisms. The antioxidant mechanisms include inhibition of ROS sources, attenuation of the AGE/RAGE–ROS axis, activation of Nrf2/ARE-mediated antioxidant defense, and protection of mitochondrial function. The anti-inflammatory mechanisms include modulation of NF-κB signaling, MAPK/JNK-mediated stress–inflammatory signaling, and gut microbiota–intestinal barrier regulation. These pathways are interconnected components of an integrated redox–inflammatory network rather than independent mechanisms. The potential outcomes mainly include improved metabolic biomarkers, reduced oxidative stress and inflammation, β-cell and tissue protection, and delayed progression of diabetic complications. Created with BioGDP.com [11]. Arrows indicate proposed regulatory relationships within the integrated network; bidirectional arrows indicate crosstalk between antioxidant and anti-inflammatory mechanisms. Blue denotes antioxidant mechanisms, purple denotes anti-inflammatory mechanisms, and the lower panel summarizes potential outcomes; colors do not indicate evidence strength.

In summary, curcumin regulates oxidative stress in T2DM through multiple interconnected mechanisms, including reduced ROS generation, attenuation of AGE/RAGE-mediated oxidative–inflammatory amplification, activation of Nrf2/ARE antioxidant defense, preservation of mitochondrial function, and protection of pancreatic β-cells and insulin signaling. Among these mechanisms, reductions in ROS production, lipid peroxidation, antioxidant enzyme depletion, mitochondrial injury, and β-cell oxidative damage are supported mainly by cell culture and animal studies, with partial clinical support from changes in circulating oxidative stress biomarkers. Direct pathway-level validation in human T2DM target tissues remains limited. These mechanisms are schematically integrated with inflammatory regulatory pathways in Figure 2, and representative evidence is summarized in Table 1.

## 6. Molecular Mechanisms Underlying Curcumin Regulation of Chronic Inflammation in Type 2 Diabetes Mellitus

Chronic low-grade inflammation is a fundamental pathological basis for T2DM development and complication progression. Long-term hyperglycemia, hyperlipidemia, insulin resistance, and metabolic tissue stress drive sustained inflammatory cytokine release and immune cell infiltration in adipose tissue, liver, pancreatic islets, kidney, and myocardium, which in turn worsen insulin signaling impairment, β-cell decline, and target organ damage. Available evidence shows that curcumin and its formulations inhibit NF-κB, MAPK/JNK, and other pro-inflammatory signaling pathways, reduce inflammatory markers including TNF-α, IL-1β, IL-6, and CRP, and ameliorate inflammation in select metabolic tissues. This section focuses on NF-κB signaling, the MAPK/JNK pathway, metabolic tissue inflammation, and gut-derived low-grade inflammation; areas with less robust evidence such as the NLRP3 inflammasome and macrophage M1/M2 polarization are discussed cautiously as mechanistic extensions. Because oxidative and inflammatory pathways overlap extensively, this section emphasizes inflammatory outputs and immune–metabolic amplification, including cytokine production, NF-κB and MAPK/JNK activation, tissue inflammation, macrophage-related responses, and gut-derived metabolic endotoxemia. Pathways introduced in the oxidative stress section are discussed here from the perspective of inflammatory propagation rather than repeated as independent oxidative mechanisms.

### 6.1. Inhibition of Inflammatory Cytokine Release and NF-κB Signaling

NF-κB is a key transcriptional regulator of chronic inflammation in T2DM. Hyperglycemia, hyperlipidemia, oxidative stress, and AGE/RAGE signaling induce NF-κB activation and promote the release of TNF-α, IL-1β, IL-6, and other pro-inflammatory cytokines, thereby aggravating β-cell damage, insulin resistance, and diabetes-related tissue inflammation. Inhibition of NF-κB signaling is one of the best-supported mechanisms underlying the anti-inflammatory effects of curcumin.

In STZ/high-fat diet-induced T2DM rats, curcumin intervention reduced fasting blood glucose and promoted pancreatic functional recovery, while alleviating pancreatic tissue destruction and apoptosis. Further analysis revealed that curcumin decreased IL-1β, IL-6, TNF-α, caspase-3, Bax, and MDA levels and increased Bcl-2, SOD2, and GPx. Mechanistically, curcumin suppressed JNK and NF-κB phosphorylation, blocking the RAGE/JNK/NF-κB signaling axis and reducing β-cell inflammation and apoptosis [62]. These findings suggest that in the setting of β-cell injury, curcumin reduces pro-inflammatory cytokine release and preserves islet structure and function, at least in part via inhibition of NF-κB-dependent inflammatory signaling.

Studies of diabetic cardiomyopathy also support NF-κB-dependent anti-inflammatory effects of curcuminoids. The curcumin analog JM-2 improved cardiac function and structural damage and reduced myocardial inflammation and fibrosis in a diabetic cardiomyopathy model. Mechanistically, JM-2 inhibited cardiac NF-κB activation and blunted pro-inflammatory cytokine elevation and macrophage infiltration [70]. Since this study employed a curcumin analog rather than native curcumin, the evidence supports the conclusion that curcuminoids target NF-κB inflammatory signaling, but cannot be directly extrapolated to unformulated curcumin across all T2DM contexts.

Clinical studies indicate that curcumin improves systemic inflammatory status in T2DM patients. A meta-analysis of 18 clinical trials including 1382 T2DM patients found that curcumin supplementation significantly reduced fasting blood glucose, HbA1c, and C-reactive protein, indicating anti-inflammatory activity alongside glycemic improvement [31]. A separate randomized double-blind placebo-controlled trial found that 1500 mg/day curcumin for 10 weeks reduced high-sensitivity C-reactive protein and triglycerides and increased adiponectin in T2DM patients, suggesting curcumin may modulate diabetic complication risk via improved inflammation and lipid metabolism [71]. That said, most clinical studies have relied on peripheral inflammatory markers such as CRP, TNF-α, and IL-6 as endpoints, with few direct assessments of NF-κB activation status. Thus, evidence for direct NF-κB inhibition by curcumin in human T2DM remains incomplete. In addition, NF-κB should not be viewed as an exclusively pathological pathway, because it also participates in immune defense, cell survival, and tissue repair. Therefore, the therapeutic implication of curcumin should be interpreted as restoration of inflammatory balance under chronic metabolic stress rather than complete or non-selective suppression of NF-κB-dependent immune responses.

### 6.2. Regulation of MAPK/JNK-Mediated Stress–Inflammatory Signaling

The MAPK/JNK pathway is a critical signaling node linking inflammation and cellular stress in T2DM. Activation by hyperglycemia, hyperlipidemia, ROS, and AGEs/RAGE drives JNK and p38-MAPK pathway activation, inducing pro-inflammatory cytokine expression, apoptosis, and insulin signaling impairment. Compared with mechanisms such as the NLRP3 inflammasome, evidence for curcumin modulation of the MAPK/JNK pathway in T2DM is more direct, making it an important component of its anti-inflammatory mechanism.

In a pancreatic injury model of STZ/high-fat diet-induced T2DM rats, curcumin blocked the RAGE/JNK/NF-κB signaling cascade by inhibiting JNK and NF-κB phosphorylation, accompanied by reduced IL-1β, IL-6, TNF-α, and apoptosis-related molecules. This indicates that JNK/NF-κB signaling is a key mechanism by which curcumin alleviates β-cell inflammatory damage [62]. These findings suggest that curcumin does not act on isolated inflammatory factors, but rather modulates RAGE-mediated stress–inflammatory signaling cascades. Because MAPK/JNK signaling can be activated by both ROS-dependent oxidative stress and cytokine-driven inflammatory stimulation, it is better interpreted as a bridge between redox imbalance and inflammatory amplification in T2DM.

In HFD/STZ-induced T2DM rats, curcumin nanoparticles alleviated oxidative stress and inflammation in the liver and pancreas. Hepatic inflammation induced by HFD/STZ was associated with increased phosphorylation of p38-MAPK pathway molecules, and nanoparticle intervention reduced aberrant p38-MAPK phosphorylation while restoring suppressed AKT signaling [72]. Given that this study used nanoformulated curcumin and delivery system effects may contribute, the findings support the view that curcumin nanoparticles may modulate hepatic and pancreatic inflammation in T2DM via downregulation of the MAPK stress pathway, but cannot be generalized to all curcumin formulations.

The AGE-RAGE pathway also promotes diabetic inflammatory damage through downstream NF-κB and MAPK/JNK signaling. In HFD/STZ-induced T2DM mice with liver injury, curcumin improved hyperglycemia, reduced hepatic oxidative stress and inflammation, and inhibited the AGE-RAGE pathway and its downstream PI3K/Akt and NF-κB signals, suggesting it mitigates diabetic liver injury by blocking glycation stress-related inflammatory pathways [51]. Taken together, MAPK/JNK and NF-κB form an integrated signaling network through which curcumin regulates inflammatory damage in T2DM.

### 6.3. Modulation of Metabolic Tissue Inflammation and Macrophage Infiltration

In T2DM and obesity, metabolic tissues, including adipose tissue, myocardium, kidney, and liver, exhibit immune cell infiltration and inflammatory cytokine release. Macrophage infiltration is a hallmark of metabolic tissue inflammation and contributes to insulin resistance, tissue fibrosis, and functional decline via the release of TNF-α, IL-6, IL-1β, MCP-1, and other mediators. Other immune cells, including T lymphocytes and neutrophils, may also participate in chronic metabolic inflammation associated with insulin resistance, although their modulation by curcumin in T2DM remains less well characterized. Current evidence supports that curcumin or curcuminoids reduce metabolic tissue inflammation and macrophage infiltration, but is insufficient to confirm systematic regulation of macrophage M1/M2 polarization.

In models of obesity-related metabolic inflammation, dietary curcumin improved glucose tolerance, insulin tolerance, and HbA1c in high-fat diet-induced obese mice and ob/ob mice. It also reduced macrophage infiltration in white adipose tissue, increased adipose tissue adiponectin production, and decreased hepatic NF-κB activity, hepatic inflammatory markers, and hepatomegaly [66]. This study supports that curcumin improves obesity-related metabolic abnormalities by reducing adipose and hepatic inflammation, but its primary endpoints are reduced macrophage infiltration and lower inflammatory cytokines; it cannot support a conclusion that curcumin directly drives macrophage phenotypic switching.

In diabetic cardiomyopathy models, the curcumin analog JM-2 reduced myocardial pro-inflammatory cytokine elevation and macrophage infiltration, and improved myocardial inflammation, fibrosis, and cardiac dysfunction, effects associated with NF-κB inhibition [70]. In diabetic kidney disease research, the curcumin derivative dimethoxycurcumin reduced the urinary albumin/creatinine ratio in high-fat diet-induced diabetic mice and improved glomerular ultrastructure and inflammatory signaling. In vitro, dimethoxycurcumin inhibited inflammatory signaling and macrophage migration induced by high glucose or palmitic acid plus LPS, and reduced NADPH oxidase 2/4 expression and oxidative DNA damage [73].

Overall, the evidence supports the conclusion that curcumin and its derivatives may improve the inflammatory microenvironment of adipose tissue, myocardium, and kidney by reducing macrophage infiltration in metabolic tissues, lowering pro-inflammatory cytokine expression, and inhibiting NF-κB and other inflammatory signals.

### 6.4. Regulation of Gut Microbiota, Intestinal Barrier, and Metabolic Endotoxemia

Gut microbiota dysbiosis, intestinal mucosal barrier disruption, and metabolic endotoxemia are important contributors to chronic low-grade inflammation in T2DM. High-fat diets and glucose and lipid metabolism disturbances alter gut microbial composition and compromise intestinal barrier integrity, allowing bacterial products to enter the circulation and trigger systemic low-grade inflammation. Orally administered curcumin has low systemic bioavailability but interacts bidirectionally with the gut microbiota locally in the intestine, making the gut a potentially important site for its antidiabetic and anti-inflammatory actions [74].

Studies in HFD/STZ-induced T2DM rats provide direct preclinical evidence. Curcumin improved intestinal integrity, hyperglycemia, and insulin resistance in diabetic rats, and markedly suppressed high-fat diet-induced metabolic endotoxemia. Additionally, curcumin reversed high-fat diet-induced gut microbiota dysbiosis, protected the intestinal mucosal barrier, improved insulin resistance, and lowered blood glucose [68]. Thus, curcumin may participate in T2DM regulation via the pathway “improved gut microbiota and intestinal barrier → reduced metabolic endotoxemia → attenuated low-grade inflammatory load”. This pathway represents a biologically plausible gut-mediated anti-inflammatory mechanism supported mainly by preclinical evidence.

Studies of curcumin-containing dietary fiber interventions show that composite dietary fiber formulas alter gut microbiota composition in T2DM mice, with curcumin-supplemented formulas influencing shifts in taxa including Akkermansia muciniphila, Faecalibaculum, and Bifidobacterium—changes correlated with improved glucose and lipid metabolism [68]. These findings suggest complex interactions among curcumin, dietary matrix, gut microbiota, and microbial metabolites. Although these findings support a gut-mediated anti-inflammatory mechanism in experimental T2DM models, human microbiome evidence remains scarce. At present, causality between curcumin-induced microbiota changes and clinical metabolic improvement in T2DM cannot be established. Therefore, the translational implications of microbiota modulation by curcumin should be interpreted cautiously. Future investigations integrating metagenomics, metabolomics, intestinal barrier function assessment, and clinical metabolic endpoints are needed to establish reproducibility and causality in human populations.

Taken together, the mechanistic evidence discussed in Section 5 and Section 6 indicates that curcumin may regulate multiple oxidative and inflammatory pathways relevant to T2DM, including AGE/RAGE, Keap1/Nrf2/ARE, mitochondrial stress, NF-κB, MAPK/JNK, and gut-derived inflammatory signaling. However, the strength of evidence differs substantially across mechanisms. Direct pathway-level evidence is strongest in cell culture and animal models, whereas human studies mainly provide indirect support through changes in circulating oxidative stress and inflammatory biomarkers. Therefore, these mechanisms should be interpreted as experimentally supported but not yet fully clinically validated pathways. This multi-target regulatory framework is summarized in Figure 2, and representative preclinical evidence is summarized in Table 1.

## 7. Clinical Evidence, Complication Protection, and Translational Outlook of Curcumin for Type 2 Diabetes Mellitus

### 7.1. Clinical Intervention Evidence

#### 7.1.1. Summary of Clinical Evidence from Randomized Trials and Meta-Analyses

Research on curcumin for T2DM has progressed from cellular and animal experiments to randomized controlled trials, systematic reviews, and formulation development studies. Available clinical evidence suggests that curcumin or curcuminoid formulations may improve fasting blood glucose, HbA1c, insulin resistance, inflammatory markers, and selected cardiometabolic and hepatic risk parameters to varying degrees, but efficacy is strongly influenced by study population, background therapy, dosage, intervention duration, formulation type, and bioavailability. The major randomized controlled trials, systematic reviews, and meta-analyses supporting these clinical observations are summarized in Table 2.

A randomized, double-blind, placebo-controlled trial enrolling 272 T2DM patients found that 1500 mg/day curcumin for 12 months resulted in lower fasting blood glucose and HbA1c compared with placebo, accompanied by increased HOMA-β, decreased HOMA-IR, higher adiponectin, lower leptin, and reduced BMI [75]. Systematic reviews and meta-analyses likewise confirm that curcumin supplementation reduces fasting blood glucose, HbA1c, and C-reactive protein, with interventions longer than 12 weeks more likely to yield glycemic improvements [31,39].

Dose–response meta-analysis shows that curcumin or turmeric supplementation reduces fasting blood glucose, HbA1c, fasting insulin, HOMA-IR, and oral glucose tolerance test glucose levels, with dosage and formulation type likely influencing efficacy [37]. However, inconsistent benefits have been reported in some studies, underscoring that plain turmeric powder, curcumin extracts, nano-curcumin, and curcumin–piperine combinations are not interchangeable. Clinical conclusions must be interpreted in the context of formulation characteristics, intervention duration, and population background.

#### 7.1.2. Critical Appraisal and Sources of Clinical Heterogeneity

Although available clinical studies and recent meta-analyses suggest that curcumin or curcuminoid formulations may improve selected metabolic, inflammatory, and oxidative stress-related biomarkers in T2DM, these findings should be interpreted with caution. Most available trials are limited by modest sample sizes, relatively short or variable intervention durations, heterogeneous curcumin formulations, inconsistent bioavailability, and differences in background antidiabetic therapy [37]. In addition, many studies primarily rely on surrogate biochemical endpoints, including fasting blood glucose, HbA1c, HOMA-IR, CRP, TNF-α, IL-6, MDA, TAC, SOD, GPx, and GSH, rather than clinically meaningful long-term outcomes.

The heterogeneity of clinical findings may be partly explained by differences in curcumin dose, formulation type, treatment duration, baseline metabolic status, disease severity, inflammatory burden, concomitant medications, and patient adherence [80]. Conventional curcumin powder, curcumin extracts, curcumin–piperine combinations, nano-curcumin, micellar preparations, liposomal formulations, and phytosomal preparations should not be considered interchangeable, because they may differ substantially in solubility, absorption, metabolism, systemic exposure, and tissue distribution [42]. Therefore, positive findings obtained with one formulation cannot be directly extrapolated to all curcumin-based interventions.

Several factors may help explain why some clinical studies report favorable outcomes whereas others show neutral or inconsistent results. First, the administered dose may not accurately reflect systemic or tissue exposure because of differences in absorption, metabolism, and formulation-dependent bioavailability. Second, patients with prediabetes, early T2DM, poorly controlled diabetes, obesity, MASLD, diabetic kidney disease, or established microvascular complications may differ in oxidative stress burden, inflammatory status, residual β-cell function, and background medication use. Third, intervention duration varies across trials, and short-term supplementation may be sufficient to alter circulating biomarkers but insufficient to affect HbA1c, organ injury, or complication-related outcomes [80]. Finally, concomitant therapies, including metformin, insulin, statins, antihypertensive drugs, SGLT2 inhibitors, and GLP-1 receptor agonists, may modify baseline metabolic and inflammatory status and may obscure or amplify the apparent effects of curcumin.

Importantly, current clinical evidence mainly supports potential improvements in glycemic parameters and inflammatory–oxidative stress biomarkers, whereas evidence demonstrating reductions in long-term diabetic complications, cardiovascular events, renal disease progression, mortality, or quality of life remains insufficient [32]. Accordingly, curcumin should be considered a potential adjunctive intervention candidate rather than an established disease-modifying therapy for T2DM. Future studies should be larger, longer, and better standardized, with predefined formulation characteristics, pharmacokinetic evaluation, clinically meaningful endpoints, and careful assessment of background therapy.

#### 7.1.3. Translational Interpretation of Clinical Effect Size and Consistency

From a translational perspective, the currently reported clinical effects of curcumin should be interpreted primarily as modest biomarker-level improvements rather than established disease-modifying outcomes. Across meta-analyses, the most consistent signals are observed for fasting blood glucose, HbA1c, HOMA-IR, CRP, TNF-α, IL-6, MDA, GSH, and TAC, suggesting that curcumin may exert measurable metabolic and redox–inflammatory activity in selected populations [31,32,37,38,39]. For example, one meta-analysis of 18 randomized trials involving 1382 T2DM patients reported reductions in fasting blood glucose, HbA1c, and CRP [31], whereas a recent dose–response meta-analysis in prediabetes and T2DM reported favorable changes in fasting blood glucose, HbA1c, fasting insulin, HOMA-IR, and OGTT glucose [37].

However, the magnitude of these effects remains modest compared with contemporary glucose-lowering pharmacotherapy, and the clinical significance of such biomarker changes remains uncertain in the absence of long-term outcome data [25,26,27,33,34]. Improvements in glycemic, inflammatory, or oxidative stress markers do not by themselves establish prevention of diabetic complications, cardiovascular events, kidney disease progression, neuropathy progression, retinopathy progression, mortality, or quality-of-life impairment [31,32,37,38,39]. Therefore, curcumin should be interpreted as a potential adjunctive intervention candidate rather than a clinically established disease-modifying treatment for T2DM.

The available evidence also remains insufficient to determine whether redox–inflammatory improvements are independent of improvements in glycemia, body weight, adiposity, hepatic fat, or background pharmacotherapy. Patients with higher baseline inflammatory or oxidative stress burden, obesity, MASLD, insulin resistance, or poor metabolic control may theoretically be more responsive, but this hypothesis requires prospective validation [5,7,48,75]. Similarly, although some meta-analyses suggest dose- or formulation-dependent effects, heterogeneous preparations and incomplete pharmacokinetic reporting prevent firm conclusions regarding dose–response relationships [32,37,80]. Future trials should therefore evaluate standardized curcumin formulations as adjuncts to evidence-based therapy and should include predefined redox–inflammatory endpoints, pharmacokinetic assessment, and clinically meaningful outcomes.

### 7.2. Protective Effects Against Chronic Complications

Chronic complications of T2DM involve multi-organ damage affecting the liver, cardiovascular system, kidney, nerves, eyes, and skin. Their shared pathological bases include long-term hyperglycemia, insulin resistance, oxidative stress, chronic inflammation, endothelial dysfunction, and tissue fibrosis. However, the strength of evidence differs substantially across T2DM-related complications. At present, clinical evidence is relatively stronger for MASLD-related hepatic and metabolic biomarkers and selected ASCVD risk-related markers, whereas evidence for diabetic nephropathy, retinopathy, neuropathy, and wound healing remains limited, preliminary, or inconsistent. Importantly, preclinical organ protection should not be equated with demonstrated prevention or treatment of human diabetic complications. Therefore, the evidence strength across T2DM-related complications is summarized in Table 3.

As summarized in Table 3, curcumin-related benefits in T2DM complications are currently supported mainly by surrogate biomarkers, selected risk markers, or preclinical organ-protective findings. Therefore, these findings should not be interpreted as definitive evidence that curcumin prevents or treats long-term human diabetic complications.

#### 7.2.1. Diabetic Cardiovascular and Hepatic Complications

In T2DM patients with MASLD, curcumin has shown potential benefits for selected hepatic, metabolic, inflammatory, and oxidative stress-related biomarkers. Randomized controlled trials show that 1500 mg/day curcumin for 12 months reduced TNF, IL-1β, IL-6, and MDA levels, increased GPx and SOD activity, and lowered non-esterified fatty acids, body fat, BMI, hepatic steatosis, and liver stiffness [48]. Another study in obese T2DM patients with MASLD likewise reported reduced hepatic fat content, liver stiffness, and HbA1c, alongside improved inflammatory and oxidative stress markers [7]. However, these findings should be interpreted as improvements in surrogate hepatic and metabolic markers rather than definitive evidence that curcumin prevents long-term MASLD progression or liver-related clinical outcomes in T2DM.

Regarding cardiovascular risk, curcumin improves select ASCVD risk-related markers but has not been shown to reduce hard cardiovascular endpoints. Studies indicate that add-on curcumin to conventional therapy reduces blood pressure, LDL-C, TNF-α, and MDA, increases HDL-C, and improves ASCVD risk stratification; however, no significant intergroup differences were observed for HbA1c or fasting blood glucose [76]. Accordingly, its value is best framed as improvement of selected risk factors and inflammatory–oxidative stress markers.

#### 7.2.2. Diabetic Nephropathy

Diabetic nephropathy is a microvascular complication characterized by albuminuria, declining renal function, oxidative stress, inflammation, and progressive renal injury. Clinical evidence on curcumin in diabetic kidney disease has been summarized in a systematic review and meta-analysis of randomized, double-blind, placebo-controlled trials, in which the reported outcomes mainly included renal function parameters, proteinuria-related indices, lipid profile, blood pressure, and fasting glucose [81]. In addition, a small randomized, double-blind clinical trial in 46 patients with T2DM and overt albuminuria reported that curcumin attenuated macroscopic proteinuria when used as an adjunctive intervention [82]. However, available studies remain insufficient to establish whether curcumin slows long-term eGFR decline, prevents end-stage kidney disease, or reduces kidney-related mortality in patients with T2DM.

#### 7.2.3. Diabetic Retinopathy and Neuropathy

Diabetic retinopathy and neuropathy are closely related to oxidative stress, microvascular injury, inflammation, mitochondrial dysfunction, and tissue-specific cellular damage. In diabetic retinopathy, preclinical studies have shown that curcumin can modulate diabetes-induced retinal oxidative stress and inflammatory changes in animal models [83,89]. More recently, a randomized controlled trial evaluated curcumin–piperine supplementation in patients with nonproliferative diabetic retinopathy using laboratory parameters and OCT/OCTA-based retinal outcomes [84]. However, current clinical evidence remains limited and is not sufficient to conclude that curcumin prevents retinopathy progression or improves long-term vision-related outcomes.

For diabetic peripheral neuropathy, clinical findings are limited and not fully consistent. One randomized, double-blind, placebo-controlled trial reported that nano-curcumin supplementation reduced diabetic sensorimotor polyneuropathy severity and improved some glycemic parameters [85]. In contrast, a more recent randomized, double-blind clinical trial found that 16-week nanocurcumin supplementation did not improve pain, neuropathic outcomes, or metabolic/cardiovascular parameters in patients with T2DM and diabetic peripheral neuropathy [86]. Therefore, the potential benefit of curcumin in diabetic neuropathy remains uncertain and requires further validation in larger, well-designed clinical studies.

#### 7.2.4. Wound Healing and Other Related Complications

Impaired wound healing and diabetic foot ulcer involve persistent inflammation, oxidative stress, endothelial dysfunction, impaired angiogenesis, and defective tissue repair. A randomized, double-blind, placebo-controlled trial in patients with grade 3 diabetic foot ulcer evaluated 80 mg/day nanocurcumin for 12 weeks and reported improvements in glycemic control, insulin resistance, lipid parameters, TAC, and GSH, but did not show significant improvement in ulcer-size indicators [87]. In animal models, a preclinical systematic review and meta-analysis found that curcumin was associated with improved wound healing rate and blood vessel density, whereas its anti-inflammatory effects in diabetic wounds remained controversial [88]. Taken together, these findings suggest that curcumin-related wound-healing effects remain preliminary, and further clinical studies are required to define optimal formulation, route of administration, dose, treatment duration, and clinically meaningful wound-healing endpoints.

### 7.3. Formulation Optimization, Bioavailability, Safety, Drug Interactions, and Combination Therapy

A core barrier to clinical translation of curcumin is its low bioavailability. Its high lipophilicity, poor aqueous solubility, chemical instability, limited intestinal absorption, rapid metabolism, and fast systemic clearance result in low effective exposure after oral administration of unformulated curcumin [90]. Technologies including nanoparticles, liposomes, nanomicelles, solid lipid nanoparticles, nanoemulsions, self-emulsifying delivery systems, protein carriers, and chitosan nanocarriers have been applied to enhance the solubility, stability, intestinal absorption, and tissue delivery of curcumin [41,43,91].

A further translational challenge is that improved pharmacokinetic exposure does not necessarily guarantee superior clinical efficacy [92]. Conventional curcumin powder, curcumin extracts, curcumin–piperine combinations, phytosomal curcumin, nano-curcumin, micellar preparations, liposomal formulations, and other enhanced-bioavailability products differ substantially in solubility, absorption, metabolism, systemic exposure, and tissue distribution [41,44]. Therefore, findings obtained with one formulation cannot be directly extrapolated to all curcumin-based interventions. This formulation-related heterogeneity may partly explain inconsistent clinical outcomes across trials [38,39]. These formulation-dependent differences are central to clinical translation, because the administered dose alone cannot represent systemic exposure, tissue distribution, or clinical comparability. Major formulation-dependent translational considerations are summarized in Table 4.

As summarized in Table 4, formulation-centered interpretation is critical because the nominal administered dose does not necessarily reflect systemic or tissue exposure. Therefore, a dose of “500 mg curcumin” cannot be assumed to be equivalent across conventional extract, piperine-enhanced, nano-based, micellar, liposomal, or phytosomal preparations. Pooling heterogeneous formulations may obscure true efficacy, underestimate formulation-specific benefits, or overgeneralize findings from one preparation to all curcumin-based interventions. Future trials should therefore report curcuminoid content, formulation technology, absorption enhancers, pharmacokinetic parameters, background therapy, safety monitoring, and clinically meaningful endpoints.

Standardization also remains a major obstacle. Commercially available curcumin products may differ in curcuminoid content, purity, excipients, absorption enhancers, release characteristics, and quality-control standards [80,97]. In particular, products containing absorption enhancers such as piperine should be distinguished from conventional curcumin preparations, because piperine may alter intestinal absorption and drug metabolism and may therefore influence both efficacy and interaction risk. Moreover, dose equivalence between conventional curcumin and enhanced-bioavailability formulations is difficult to define, because the administered dose may not reflect systemic or tissue exposure. Future clinical studies should clearly report formulation characteristics, curcuminoid content, absorption enhancers, pharmacokinetic parameters, treatment duration, and background therapy. Ideally, standardized formulations with validated bioavailability and reproducible quality control should be evaluated in adequately powered trials with clinically meaningful endpoints.

Overall, available short-term human studies suggest that oral curcumin is generally well tolerated, but its safety profile should not be overgeneralized. Long-term administration, high-dose supplementation, and enhanced-bioavailability formulations require further safety evaluation. In human studies, oral curcumin at 6 g/day for 4–7 weeks showed no overt toxicity, but gastrointestinal adverse events were reported [98]. In T2DM clinical trials, curcumin has generally been well tolerated, with mild gastrointestinal discomfort being the most commonly reported adverse event [7,75]. Potential drug–drug interactions should also be considered, particularly because patients with T2DM frequently receive multiple medications, including glucose-lowering agents, antihypertensive drugs, lipid-lowering drugs, antiplatelet agents, and anticoagulants. Interactions with anticoagulant or antiplatelet medications may be clinically relevant and should be evaluated cautiously, especially in patients with cardiovascular disease or polypharmacy [99]. Enhanced-bioavailability formulations and curcumin–piperine combinations may also require separate safety evaluation, because increased systemic exposure or altered metabolism may influence tolerability and interaction risk [93]. Regarding combination therapy, curcumin–piperine combinations have been reported to improve selected metabolic markers by enhancing curcumin bioavailability [8,77]. Future studies should also evaluate curcumin as an adjunct to established first-line therapies, including metformin, SGLT2 inhibitors, and GLP-1 receptor agonists, with particular attention to potential additive effects on oxidative stress, inflammation, insulin resistance, and long-term diabetic complications. However, evidence for curcumin combined with conventional glucose-lowering drugs remains limited and is best regarded as an exploratory research direction at present.

In summary, curcumin intervention may improve selected markers of glucose metabolism, insulin resistance, inflammatory status, and cardiometabolic or hepatic risk in T2DM, but low bioavailability, formulation heterogeneity, limited standardization, potential drug–drug interactions, and insufficient long-term safety data remain important translational bottlenecks. Future studies should employ standardized formulations, well-defined dosages, and adequate treatment durations, and integrate glycemic control, inflammatory–oxidative stress markers, pharmacokinetics, safety monitoring, target organ indices, and long-term clinical outcomes within a unified study framework.

## 8. Conclusions and Discussion

T2DM is a chronic metabolic disease driven collectively by insulin resistance, pancreatic β-cell functional decline, dysregulated glucose and lipid metabolism, and multi-organ damage [100]. Long-term hyperglycemia and hyperlipidemia persistently induce oxidative stress and chronic low-grade inflammation, further exacerbating impaired insulin signaling, β-cell damage, and the development of diabetes-related complications [101,102]. Current glucose-lowering therapies have advanced glycemic control, weight management, and cardiorenal protection, but direct interventions targeting oxidative stress and inflammatory responses remain relatively limited.

Curcumin possesses diverse pharmacological activities including antioxidant, anti-inflammatory, lipid-regulating, insulin-sensitizing, and tissue-protective effects [53]. Accumulating evidence indicates that curcumin alleviates T2DM-associated oxidative stress by inhibiting ROS production, reducing NADPH oxidase activity, modulating the AGE/RAGE axis, activating the Keap1/Nrf2/ARE antioxidant defense system, improving mitochondrial function, and reducing lipid peroxidation damage [45,47]. Meanwhile, it lowers inflammatory cytokine levels and ameliorates metabolic tissue inflammation by suppressing NF-κB, MAPK/JNK, and other inflammation-related signaling pathways, and may improve gut-derived low-grade inflammation by regulating gut microbiota and intestinal barrier function [101,103,104].

In terms of overall mechanism, curcumin is not a single-target hypoglycemic agent. Its antidiabetic effects are best understood as a multi-target regulatory process centered on antioxidant and anti-inflammatory actions, with secondary benefits for glucose and lipid metabolism and target organ protection [44,96]. Preclinical and selected clinical studies suggest improvements in β-cell damage, insulin signaling impairment, hepatic lipid deposition, inflammatory cytokines, and oxidative stress markers, but the evidence base remains constrained by sample size, intervention duration, dosage, formulation type, bioavailability, and population heterogeneity. Moreover, most clinical studies have focused on surrogate biochemical markers rather than long-term clinical outcomes, and there is currently insufficient evidence to conclude that curcumin reduces diabetic complications, cardiovascular events, mortality, or quality-of-life impairment in T2DM.

Overall, curcumin shows promise as a multi-target adjunctive intervention candidate for T2DM, particularly through modulation of oxidative stress, chronic inflammation, insulin resistance, and MASLD-related metabolic abnormalities. However, the distinction between promising preclinical mechanisms and more limited clinical validation should be emphasized. Current clinical evidence most consistently supports improvements in inflammatory and oxidative stress biomarkers, insulin resistance-related indices, and selected hepatic metabolic outcomes, whereas evidence for preventing long-term diabetic complications, cardiovascular events, kidney disease progression, mortality, or quality-of-life impairment remains insufficient.

Before curcumin can be considered for routine clinical application, standardized formulations and dosing strategies, pharmacokinetic evaluation, long-term safety assessment, and large multicenter randomized controlled trials are essential. Future studies should also explore biomarker-guided or precision medicine approaches to identify patient subgroups with pronounced oxidative stress or inflammatory phenotypes who may be more likely to benefit. In this context, curcumin should be viewed not as a replacement for established antidiabetic therapy, but as a potential component of an integrated T2DM management strategy combining lifestyle modification, evidence-based pharmacotherapy, and selected adjunctive interventions.

## Figures and Tables

**Table 1:** Representative mechanistic evidence for curcumin in modulating oxidative stress and chronic inflammation in T2DM.

Experimental Model	Target Pathway(s)	Key Molecular Findings	Tissue/Organ	Evidence Level	Reference
STZ + HFD rat (Standard T2DM model)	[Inf] NF-κB/JNK ↓ [Inf] Cytokines ↓ [β] β-cell apoptosis ↓	IL-1β ↓, IL-6 ↓, TNF-α ↓ Caspase-3 ↓, Bax ↓, MDA ↓ SOD2 ↑, GPx ↑	Pancreas/Islets	Animal study	[62]
INS-1 cells + STZ rat (High glucose/palmitate)	[OS] NADPH oxidase ↓ [β] β-cell survival ↑	ROS ↓, NADPH oxidase subunits ↓ Insulin secretion ↑, Apoptotic factors ↓	Pancreatic β-cells	Cell, animal	[52]
T2DM rat (HFD + STZ) (Nrf2-focused)	[Nrf2] Keap1/Nrf2/ARE activation [OS] Lipid peroxidation ↓	MDA ↓, GPx ↑, SOD ↑, CAT ↑ Nrf2 nuclear translocation ↑ NQO1 ↑, HO-1 ↑	Kidney/Testis	Animal study	[55,56]
HFD/STZ mouse (Hepatic targeting)	[OS] AGE–RAGE axis ↓[Inf] NF-κB ↓[β] PI3K/Akt ↑	AGE-RAGE ↓, NF-κB ↓ p-PI3K ↑, p-Akt ↑ Metabolomics profile improved	Liver	Animal study	[51]
db/db mouse (Genetic obesity T2DM)	[Mito] Mitochondrial function ↑[OS] Oxidative stress ↓[Nrf2] Nrf2 activation	Mitochondrial OCR ↑, ATPase ↑ Lipid peroxidation ↓ Abnormal NO metabolism ↓	Liver/Kidney	Animal study	[57]
STZ + HFD rat (Diabetic cardiomyopathy)	[Mito] Mitochondrial apoptosis ↓[β] Sirt1–FoxO1/PI3K–Akt ↑	MDA ↓, Cytochrome C ↓, Bax ↓ Caspase-3 ↓, Bcl-2 ↑ p-Akt ↑, FoxO1 deacetylation ↑	Myocardium	Animal study	[58,59]
Nano-curcumin + insulin, STZ rat (Diabetic kidney disease)	[Inf] NLRP3 inflammasome ↓[Inf] P38/P53/NF-κB ↓[Inf] MAPK8 ↓	NLRP3 ↓, IL-1β ↓, NF-κB ↓ Caspase-3 ↓ Glomerular basement membrane thickening ↓ Podocyte cytoskeletal impairment ↓	Kidney	Animal study	[64]
STZ rat (Diabetic nephropathy, Nrf2)	[Nrf2] Renoprotection via Nrf2[Mito] Mitochondrial redox balance	Nrf2 ↑, HO-1 ↑ Antioxidant enzymes ↑ Inflammatory response ↓, Renal dysfunction ↓	Kidney	Animal study	[65]
HFD obese mouse/ob/ob mouse (Obesity-induced insulin resistance)	[Inf] NF-κB inhibition (liver)[Inf] Macrophage M1→M2[β] Insulin signaling ↑	Hepatic NF-κB ↓, TNF-α ↓ IL-6 ↓, MCP-1 ↓ Adipose macrophage infiltration ↓ Adiponectin ↑, PPAR-γ activation ↑	Adipose tissue/Liver	Animal study	[66,67]
HFD/STZ rat (Gut–systemic inflammation axis)	[Gut] Microbiota diversity ↑[Inf] Endotoxemia ↓[Inf] TLR4/NF-κB ↓	Bacteroidetes ↑, Microbial diversity ↑ Intestinal permeability ↓, LPS ↓ TLR4 ↓, Systemic cytokines ↓ Insulin resistance ↓	Intestine/Systemic	Animal study	[68]
Animal models of diabetic cardiomyopathy (Preclinical MA; 32 studies, *n* = 681)	[OS] Myocardial oxidative stress ↓[Inf] Myocardial inflammation ↓[Mito] Autophagy ↑/Apoptosis ↓	LVEF ↑, LVFS ↑ Myocardial injury markers ↓, HW/BW ↓ Oxidative, inflammatory and apoptotic indices significantly improved (all *p* < 0.05) Dose > 200 mg/kg associated with greater efficacy	Myocardium	Preclinical systematic review	[69]

Target pathway labels: [OS] oxidative stress; [Nrf2] Nrf2/antioxidant defense; [Mito] mitochondria; [Inf] inflammatory signaling; [β] β-cell/insulin signaling; [Gut] gut microbiota. Notes: ↑, increase or upregulation; ↓, decrease or downregulation. All data are derived from in vitro or in vivo (animal) experiments; clinical translatability requires further validation in human trials. Where two references are listed for a single row, both studies contributed to the mechanistic evidence summarized. Target pathway labels in square brackets correspond to the legend above the table.

**Table 2:** Summary of major clinical evidence on curcumin supplementation in T2DM.

Study Type	Sample/Population	Intervention & Dose	Duration	Glycemic Outcomes	Inflammation/Oxidative Stress	Other Metabolic Outcomes	Adverse Events	Reference
RCT Double-blind, placebo-controlled	*n* = 272 Obese T2DM	Curcumin extract 1500 mg/day	12 months	FBG ↓ * HbA1c ↓ * HOMA-IR ↓ *** HOMA-β ↑ **	Not primary endpoint	Adiponectin ↑ *** Leptin ↓ *** BMI ↓ ***	Mild GI discomfort; well-tolerated	[75]
RCT Double-blind, placebo-controlled	*n* = 78 T2DM + MASLD	Curcumin 1500 mg/day	12 months	HbA1c ↓ ***	TNF-α ↓ *** IL-1β ↓ *** IL-6 ↓ *** MDA ↓ *** GPx ↑ *** SOD ↑ ***	Hepatic steatosis ↓ Liver stiffness ↓ NEFA ↓	Mild GI discomfort	[48]
RCT Double-blind, placebo-controlled	*n* = 227 Obese T2DM	Curcumin 1500 mg/day	12 months	HbA1c ↓ ***	IL-1β ↓ *** TNF-α ↓ *** MDA ↓ *** TAC ↑ *** GPx ↑ *** SOD ↑ ***	Liver fat ↓ Liver stiffness ↓	Mild GI discomfort; normal liver/renal function	[7]
RCT Double-blind, placebo-controlled	*n* = 114 Obese T2DM	Curcumin 1500 mg/day	12 months	FBG ↓ *** HbA1c ↓ * HOMA-IR ↓ ***	IL-6 ↓ *** IL-1β ↓ *** TNF-α ↓ *** hs-CRP ↓ * NLR ↓ * MDA ↓ *** TAS ↑ *** SOD ↑ *** GPx ↑ ***	—	Well-tolerated overall	[5]
RCT T2DM + high ASCVD risk	*n* = 72 T2DM, ASCVD risk ≥ 5%	Curcumin 500 mg tid + conventional therapy	≥3 months follow-up	HbA1c NS FBG NS	TNF-α ↓ * MDA ↓ *	SBP/DBP ↓ *** LDL-C ↓ * HDL-C ↑ * ASCVD risk ↓ **	Nausea 13.9%, headache 11.1%, diarrhea 5.6%; all mild	[76]
RCT Double-blind, placebo-controlled	*n* = 118 T2DM	Curcuminoids 1000 mg/day + piperine 10 mg/day	8 weeks	Not primary endpoint	TAC ↑ *** SOD ↑ *** MDA ↓ ***	—	No adverse events reported	[53]
RCT T2DM + hypertriglyceridemia	*n* = 72 T2DM + high TG	Curcumin 500 mg + piperine 5 mg/day	12 weeks	FBG ↓ ** HbA1c NS	CRP ↓ (marginal, *p* = 0.081)	TG ↓ ** Energy/fatigue improved *	No adverse events reported	[77]
RCT 3-arm parallel design	*n* = 60 T2DM + dyslipidemia	Curcumin 1100 mg/day + glimepiride 4 mg/day	3 months	FBG NS HbA1c NS	hs-CRP ↓ * Sirtuin-1 ↑ *	TC ↓ * TG ↓ * LDL-C ↓ * AI ↓ *		[78]
RCT Prediabetes prevention	*n* = 240 Prediabetes	Curcumin extract (dose NR)	9 months	HOMA-β ↑ ** HOMA-IR ↓ *** T2DM conversion: 0% vs. 16.4%	Adiponectin ↑ *	—	Very minor adverse effects	[79]
Meta-analysis 18 RCTs	*n* = 1382 T2DM, mean age 55.9 yr	Various formulations	Most ≥8 weeks	FBG ↓ MD = −11.48 mg/dL ** HbA1c ↓ MD = −0.54% **	CRP ↓ SMD = −0.59 *	—	High heterogeneity; interpret with caution	[31]
Meta-analysis 34 RCTs, 39 arms	Prediabetes + T2DM	Curcumin/turmeric (dose subgroups)	—	FBG ↓ WMD = −10.15 mg/dL HbA1c ↓ WMD = −0.32% HOMA-IR ↓ WMD = −0.46 Insulin ↓ WMD = −0.69 μU/mL	—	Dose ≥1 g/day and T2DM subgroup showed greater effect	Safe; substantial heterogeneity	[37]
Meta-analysis 17 RCTs, 22 arms	Metabolic disease patients	Turmeric/curcuminoids	Most ≥8 weeks	FBG ↓ WMD = −7.86 mg/dL *** HbA1c ↓ WMD = −0.38% *** HOMA-IR ↓ WMD = −1.01 *** FSI ↓ WMD = −1.69 mU/L * (>8 wk)	—	—	Significant heterogeneity noted	[38]
SR + Meta-analysis 28 RCTs, 31 effect sizes	Prediabetes + T2DM	Curcumin/turmeric (≥1 g/day more effective)	—	—	CRP ↓ SMD = −0.50 TNF-α ↓ SMD = −1.70 IL-6 ↓ SMD = −2.97 MDA ↓ SMD = −1.31 GSH ↑ SMD = 1.72 TAC ↑ SMD = 1.03	Unformulated curcumin and higher doses showed greater improvement	Low certainty of evidence; high heterogeneity	[32]
Systematic review 11 RCTs (*n* = 1131)	T2DM	Curcumin (various)	≥12 weeks more effective	FBG ↓ (8/11 studies) HbA1c ↓ (7/11 studies) HOMA-IR ↓ (3/5 studies)	—	Longer duration (≥12 wk) more consistent	Generally safe	[39]

Statistical significance: * *p* < 0.05; ** *p* < 0.01; *** *p* < 0.001. ↑, significant increase or upregulation; ↓, significant decrease or downregulation. Notes: Meta-analyses and systematic reviews represent pooled effect estimates across heterogeneous formulations and populations; results should be interpreted in conjunction with individual trial data.

**Table 3:** Evidence strength for curcumin in T2DM-related complications.

Complication	Evidence Level	Main Findings	Major Limitations	Reference
MASLD/hepatic complications	Moderate clinical evidence	RCTs reported improvements in hepatic steatosis, inflammatory markers, oxidative stress, and metabolic parameters in T2DM with MASLD	Limited long-term liver outcomes and standardized formulation data	[7,48,75]
ASCVD-related risk	Moderate clinical evidence (risk markers)	Curcumin improved selected cardiometabolic risk markers and inflammatory parameters	No evidence for reduction in cardiovascular events or mortality	[76]
Diabetic nephropathy	Limited clinical evidence + preclinical evidence	Small clinical studies suggested improvements in proteinuria-related outcomes; animal studies support renal protection	Lack of long-term renal endpoints (eGFR decline, ESKD)	[81,82]
Diabetic retinopathy	Mainly preclinical; early clinical evidence	Antioxidant and anti-inflammatory retinal effects reported; OCTA-based clinical studies remain preliminary	Insufficient evidence for preventing retinal progression	[83,84]
Diabetic neuropathy	Limited and inconsistent clinical evidence	Nanocurcumin showed potential improvement in neuropathy scores in some trials	Small sample sizes and inconsistent outcomes	[85,86]
Diabetic wound healing	Mainly preclinical + limited clinical evidence	Curcumin-related formulations improved metabolic markers and wound-related parameters in selected studies	Optimal dose, formulation, and clinical wound endpoints remain unclear	[87,88]

**Table 4:** Formulation-dependent clinical translation of curcumin interventions.

Formulation	Representative Dose/Examples	Main Translational Feature	Current Evidence	Key Limitation	References
Conventional curcumin/extract	500–1500 mg/day	Direct clinical use	Improved glycemic, inflammatory, oxidative stress, β-cell, and hepatic biomarkers	Low bioavailability; variable curcuminoid content	[7,48,75,79]
Curcumin–piperine	500–1000 mg curcuminoids + piperine	Absorption enhancement	Improved selected glycemic, lipid, inflammatory, and oxidative stress markers	Possible interaction risk; not equivalent to curcumin alone	[8,53,77,93,94]
Nanocurcumin	Commonly low-dose oral preparations; e.g., 80 mg/day	Improved dispersion and delivery	Tested in neuropathy and wound-related clinical settings	Product-specific PK; inconsistent clinical outcomes	[44,85,86,87,91]
Add-on curcumin	500 mg three times daily with standard therapy	Adjunctive use	Improved selected ASCVD risk and inflammatory–oxidative markers	No evidence for hard cardiovascular outcomes	[76]
Micellar/phytosomal/liposomal curcumin	Product-dependent	Enhanced systemic exposure	Human PK evidence supports formulation-dependent exposure	T2DM-specific outcome data remain limited	[80,92,95,96]

## Data Availability

No new data were created or analyzed in this study.

## Abbreviations

AGEs	Advanced glycation end-products
AI	Atherogenic index
Akt	Protein kinase B
ARE	Antioxidant response element
ASCVD	Atherosclerotic cardiovascular disease
Bax	Bcl-2-associated X protein
Bcl-2	B-cell lymphoma 2
BMI	Body mass index
CAT	Catalase
CRP	C-reactive protein
DPP-4	Dipeptidyl peptidase-4
ESKD	End-stage kidney disease
FBG	Fasting blood glucose
FoxO1	Forkhead box protein O1
FSI	Fasting serum insulin
GI	Gastrointestinal
GLP-1	Glucagon-like peptide-1
GLUT4	Glucose transporter type 4
GPx	Glutathione peroxidase
GSH	Glutathione
HbA1c	Glycated hemoglobin A1c
HFD	High-fat diet;
HO-1	Heme oxygenase-1
HOMA-IR	Homeostasis model assessment of insulin resistance
HOMA-β	Homeostasis model assessment of β-cell function
hs-CRP	High-sensitivity C-reactive protein
HW/BW	Heart weight-to-body weight ratio
IL-1β	Interleukin-1 beta
IL-6	Interleukin-6
IRS	Insulin receptor substrate
JNK	c-Jun N-terminal kinase
Keap1	Kelch-like ECH-associated protein 1
LDL-C/HDL-C	Low/high-density lipoprotein cholesterol
LPS	Lipopolysaccharide
LVEF	Left ventricular ejection fraction
LVFS	Left ventricular fractional shortening
MA	Meta-analysis
MACE	Major adverse cardiovascular events
MAPK	Mitogen-activated protein kinase
MASLD	Metabolic dysfunction-associated steatotic liver disease
MCP-1	Monocyte chemoattractant protein-1
MD	Mean difference
MDA	Malondialdehyde
NADPH	Nicotinamide adenine dinucleotide phosphate
NEFA	Non-esterified fatty acids
NF-κB	Nuclear factor-κB
NLR	Neutrophil-to-lymphocyte ratio
NLRP3	NLR family pyrin domain-containing protein 3
NO	Nitric oxide
NQO1	NAD(P)H quinone oxidoreductase 1
NR	Not reported
Nrf2	Nuclear factor erythroid 2-related factor 2
NS	Not significant
OCR	Oxygen consumption rate
OCTA	Optical coherence tomography angiography
PI3K	Phosphoinositide 3-kinase
PPAR-γ	Peroxisome proliferator-activated receptor gamma
p-PI3K/p-Akt	Phosphorylated PI3K/Akt
RAGE	Receptor for advanced glycation end-products
RCT	Randomized controlled trial
ROS	Reactive oxygen species
SBP/DBP	Systolic/diastolic blood pressure
SGLT2	Sodium–glucose cotransporter 2
Sirt1	Sirtuin 1
SMD	Standardized mean difference
SOD	Superoxide dismutase
SOD2	Superoxide dismutase 2
STZ	Streptozotocin
T2DM	Type 2 diabetes mellitus
TAC	Total antioxidant capacity
TAC/TAS	Total antioxidant capacity/status
TC	Total cholesterol
TG	Triglycerides
TLR4	Toll-like receptor 4
TNF-α	Tumor necrosis factor-alpha.
WMD	Weighted mean difference
